# The mean as a multilevel issue

**DOI:** 10.3389/fpsyg.2014.00180

**Published:** 2014-02-27

**Authors:** David Trafimow

**Affiliations:** Department of Psychology, New Mexico State UniversityLas Cruces, NM, USA

**Keywords:** mean, causation, multilevel causation, between-participants analyses, within-participants analyses

This General Commentary extends the excellent article by Speelman and McGann (2013) criticizing how psychology researchers often use group means when interpreting psychology research. I believe that the identified problems matter, and are part of a more general problem in psychology; the criticisms apply to more than just means.

To commence, consider a group mean. The mean might not reflect the score of any particular individual. Or consider two group means; e.g., that the mean for males exceeds the mean for females on some characteristic. This difference does not necessarily imply that the majority of males exceed the majority of females on that characteristic. Nevertheless, as Speelman and McGann noted, psychology researchers tend to draw this last type of conclusion.

The problem is part of a more general multilevel problem in psychology that can be seen easily if we make salient that researchers compute group means to support a causal hypothesis, as follows. “My hypothesis is that *X* causes *Y*, so I will manipulate *X*, and get an effect on *Y*, such that *X*_1_ gives a value of *Y*_1_ and *X*_2_ gives a value of *Y*_2_. If the means of *Y*_1_ and *Y*_2_ differ, the field will accept that a change in *X* causes a change in *Y*.” And at one level, this is perfectly reasonable. At another level, it is not. It depends on whether we are interested in causation at the group or individual level. If we are interested in causation at the group level, obtaining differences between group means is reasonable. But if the causal hypothesis is at the individual level, obtaining differences between group means might be fine as a start, but it is not a reasonable basis for a strong conclusion.

Consider, for example, the old-fashioned notion that attitudes toward performing behaviors cause people to have corresponding behavioral intentions (e.g., Fishbein and Ajzen, 1975). Theoretically, this hypothesis is at the individual level in the sense that manipulating any particular person's attitude is alleged to cause that person's behavioral intention to shift accordingly. But practically all of the performed research has been at the group level (see Fishbein and Ajzen, 2010 for a review). That is, mean behavioral intentions in either a pro or anti attitude group differ, or attitudes and behavioral intentions are correlated across a sample of individuals. After thousands of studies, there is support that attitudes cause behavioral intentions at the group level but it is not clear that this is so at the individual level. The empirical fact of a difference in the group means, or an overall correlation after statistically “controlling” for alternative explanations, fails to show that changing any particular person's attitude would cause that person's behavioral intention to change.

Suppose that an infallible Demon knows that for a given behavior and population of interest, attitudes cause behavioral intentions for 3% and that attitudes have nothing to do with behavioral intentions for the other 97%. Further suppose that a researcher performed an experiment with a large sample, using a powerful manipulation of attitudes, and found that the means differed significantly in the predicted direction. This would be interpreted as “strong support” that attitudes cause behavioral intentions even though the Demon knows that the hypothesis is wrong for 97% of the people to whom it is intended to apply! In this case, the effect is observed because of the power achieved by the large sample, and the effect on 3% gives an overall impression of an effect in the whole sample.

A within-participants design would not necessarily mitigate the problem. Suppose that a researcher measured behavioral intentions before and after manipulating participants' attitudes. The usual analysis would be to compare *before* vs. *after* means, this difference would be statistically significant provided a sufficiently large sample size, and so the natural conclusion would again be that attitudes cause behavioral intentions. In fact, the researcher might tout the use of a within-participants design as providing strong support for causation at the level of individual persons! For the use of a within-participants design to mitigate the multilevel problem, the researcher would have to perform frequency analyses, as Speelman and McGann (2013) recommended, and is rarely done. It is only in the event that frequency analyses were performed that the conclusion might approach that of our knowledgeable Demon.

The problem is not just with means. Most of the recent research connecting attitudes with behavioral intentions has been with path analyses and structural equation analyses. But changing the type of “causal” statistics does not address the multilevel problem, which is that the causal hypothesis is within-participants at the theoretical level, and between-participants at the empirical level, and the two do not correspond. Even if we ignore the usual problems with correlations, and assume that the causal analyses really do indicate causation, it is the wrong kind of causation; the fact of group level causation fails to imply corresponding individual level causation.

In conclusion, I agree with Speelman and McGann (2013) that although means can be useful, researchers tend to draw stronger conclusions from them than those that are warranted. In addition, I have attempted to demonstrate here that this is not just a problem of means, but is part of a more general failure for psychologists to recognize the difference between causation at the group level vs. at the individual level. It is possible for hypotheses to be correct at the group level and incorrect for the vast majority of individual people. Speelman and McGann complained that although their colleagues say that the problems with means are well known, they continue to commit them. I believe that the problem is a more general one of confusing causation at the group vs. individual levels. Until researchers learn to routinely distinguish between levels of causation, they will continue to make the errors that Speelman and McGann documented, as well as many additional ones. My hope is that the present General Commentary will provide an impetus in the direction of recognizing the importance of the multilevel distinction.
